# Risk Factors and Outcome of Pneumatosis Intestinalis in Children

**DOI:** 10.3390/children12020137

**Published:** 2025-01-26

**Authors:** Noha Heikal, Alessandra Mari, Jutta Köglmeier

**Affiliations:** 1 Department of Paediatric Gastroenterology, Great Ormond Street Hospital for Children NHS Foundation Trust, London WC1N 3JH, UK; noha.heikal1@nhs.net (N.H.); alessandra.mari@unimi.it (A.M.); 2 Department of Pediatrics, Ospedale dei Bambini Vittore Buzzi, University of Milan, 20154 Milano, Italy

**Keywords:** pneumatosis intestinalis, risk factors, management, prognosis, children

## Abstract

Objectives: Pneumatosis intestinalis (PI) is rare in childhood. The aetiology remains poorly understood. Our aim was to assess its associated risk factors and outcome. Methods: Retrospective data collection of all children (>1 month of age) with radiological evidence of PI identified from 1991 to 2021 in a large children’s hospital. Poor outcome was defined as loss of enteral autonomy, or death within one month of PI diagnosis. Results: A total of 31 patients (21 male, 67.7%) were included, with a median age of 5 years. The underlying diagnosis was heterogenous. Cerebral palsy and acute lymphocytic leukaemia (ALL) were most common (5/31 for each, 16.13%). A share of 12/31 (38.7%) developed PI 2–15 months post-bone marrow transplantation (BMT). Most patients (n = 15, 48.4%) had no pre-existing gastroenterological disorder. In the majority (11/31, 35.5%), PI was an incidental finding. Abdominal pain was the most common presentation in symptomatic children (7/31, 22.6%). All (31/31, 100%) were managed conservatively with gut rest and antibiotics, and 6/31 (19.4%) had a poor outcome (1/31 permanent feeding intolerance, 5/31 died). When comparing patients who did well (group 1) to those with a poor outcome (group 2), worse prognosis was associated with a lower platelet count (*p* = 0.016), raised CRP (*p* = 0.008), higher creatinine (*p* = 0.006), and higher urea (*p* = 0.013). Conclusions: The overall prognosis of PI in childhood is good but associated with significant morbidity and mortality in a small number of patients. Our data suggest that lower platelet count, and higher urea, creatinine, and CRP levels might be risk factors.

## 1. Introduction

Pneumatosis intestinalis (PI) is a radiological finding referring to the presence of air in the wall of the small and/or large intestine [1]. In newborns, mainly in preterm infants, it is a well-recognised sign of necrotizing enterocolitis (NEC). NEC is a critical condition that might lead to surgical intervention, sepsis, and death [2]. In children, both the aetiology and associated risk factors are not well understood. The true incidence is not known, as many patients have minimal symptoms at the time of diagnosis, but is estimated to be approximately 0.03% based on post-mortem studies [3]. The pathophysiology of PI remains unclear. Intestinal mucosal injury due to infection and dysbiosis, altered immune response, mechanical factors associated with pulmonary disease, and mesenteric ischaemia are the most commonly described underlying pathophysiology mechanisms [4]. Recently, PI in intestinal failure patients has been associated with the introduction of enteral feeds leading to increased gut stress [5].Clinical presentation in children varies and ranges from an incidental finding to frank rectal bleeding. Suggested risk factors include complex neurological background, malignancy, post-transplant status, and steroid use [6]. Management typically involves conservative approaches, such as gut rest and antibiotics, with a generally favourable outcome.

In this study, we investigate the clinical background, feeding pattern and symptoms at presentation, management, and outcome of children with confirmed radiological signs of PI whilst receiving inpatient therapy in a large children’s hospital in the United Kingdom. We aim to identify risk factors associated with poor outcome by comparing patients with favourable outcome to those with unfavourable outcome (death or loss of enteral autonomy).

## 2. Materials and Methods

A retrospective database review to identify all children (<18 years of age) with radiological evidence of PI between March 1991 and March 2021 at Great Ormond Street Hospital (GOSH) in London was completed. Children whose onset of PI started below the age of 4 weeks post-term were excluded to avoid inclusion of neonates with NEC. Verbal consent was obtained from parents/carers and data were anonymised. Information on demographic data, clinical presentation, feeding history, radiological and laboratory findings, management, and outcome was obtained from the electronic patient records. Poor outcome was defined as loss of enteral autonomy, or death within one month of diagnosis of PI. Comparison between the 2 groups with different outcomes was carried out to identify the risk factors of poor outcome of PI in children.

Data were analysed using the Statistical Package of Social Science (SPSS) program for Windows (Standard version 21) following discussion with the hospital statistician. The normality of data was first tested with the one-sample Kolmogorov–Smirnov test. Qualitative data are described using numbers and percentage. Association between categorical variables was tested using the Fischer exact test and Monte Carlo test when the expected cell count was less than 5. Continuous variables are presented as mean ± SD (standard deviation) for normally distributed data and median (range) for non-normal data. The two groups were compared with Student’s t-test for normal data and the Mann Whitney test for non-normal data in view of the small sample size.

## 3. Results

Thirty-one patients (21/31; 67.7% males and 10/31; 32.3% females) fulfilled the inclusion criteria. The median age at diagnosis of PI was 5 years (0.25–15 years). Most patients (12/31; 38.7%) were between 1 and 5 years old. The medical background was heterogeneous. A share of 5/31 had cerebral palsy, 5/31 ALL, and 4/31 severe combined immunodeficiency (SCID); 3/31 had received a solid organ transplantation (2/31 lung transplant, 1/31 renal transplant with colostomy in situ); 2/31 had congenital Hemophagocytic lymphohistiocytosis (HLH); and 1/31 of children with HLH also developed very early onset IBD (VEOIBD). The remaining patients had one of the following diagnosis (1/31 each): anorectal malformations (ARMs), non-Hodgkin’s lymphoma, chronic lung disease, Ewing’s Sarcoma, acute myeloid leukaemia (AML), relapsed atypical teratoid rhabdoid tumour (ATRT) of the brain and spine, WHIM (warts, hypogammaglobulinemia, infections, and myelokathexis) syndrome, dilated cardiomyopathy, chronic granulomatous disease (CGD), Emmanuel syndrome (which was associated with Hirschsprung disease), anomalous left coronary artery from the pulmonary artery (ALCAPA), and DiGeorge syndrome. Amongst these, 15/31 children developed PI following transplantation—12/31 after haematopoetic stem cell (HSCT) and 3/31 solid organ (3/31) transplantation.

The time from transplant to the development of PI ranged from 1 to 15 months (median 1 month). A share of 16/31 children developed PI whilst having gut-related pathology (see Table 1): 7/31 were transplant recipients and had developed graft versus host disease (GvHD) of the gut; 2/31 had constipation, 2/31 typhlitis, 1/31 ARM, 1/31 mucositis, 1/31 Hirschsprung disease, and 1/31 VEOIBD (3.2% for each). A share of 15/31 (48.3%) patients had no gastroenterological (GI) pathology prior to the development of PI.

**Figure 1:**
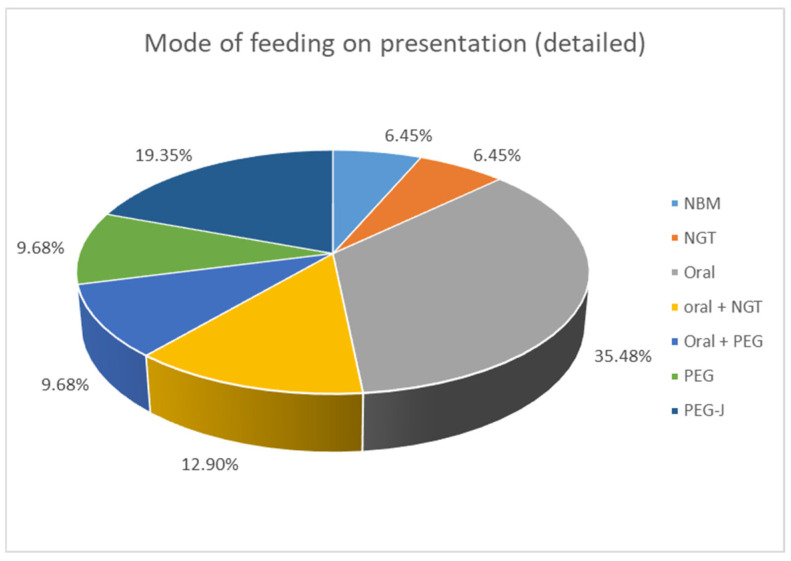
Mode of feeding at presentation of patients with PI. NBM: nil by mouth, NGT: nasogastric tube, PEG: percutaneous endoscopic gastrostomy, PEG-J: percutaneous endoscopic gastrostomy with jejunal extension.

Clinical presentation was very variable. PI was an incidental finding in 11/31 (35.5%) patients, who underwent abdominal X-ray for other reasons. In those who had clinical symptoms, 7/31 (22.6%) suffered from new onset abdominal pain. A share of 6/31 (19.4%) presented with bleeding per rectum, 4/31 (12.9%) with abdominal distention, and 4/31 (12.9%) with vomiting; 3/31 (9.6%) spiked a fever/became septic and had feed intolerance; and 2/31 (6.5%) developed diarrhoea. Some children presented with more than one symptom. The study included an assessment of the mode of feeding among the patients. When comparing enteral intake, 2/31 patients (6.4%) were already nil by mouth (NBM) when PI was diagnosed, 11/31 (35.5%) ate an age-appropriate oral diet, 7/31 (22.6%) received a combination of oral food and enteral tube feeding, and 11/31 (35.5%) were exclusively tube fed with a liquid enteral feed (see Figure 1).

**Figure 2:**
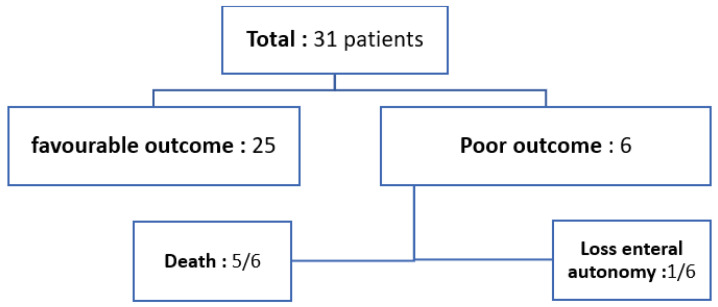
Outcome of patients with PI.

The most frequently prescribed medication at presentation was steroids, which were administered to 18/31 (58.1%) patients. A share of 20/31 (64.5%) patients underwent stool virology testing when PI was diagnosed, and 16/31 (51.6%) patients had negative stool virology results. Amongst the remaining 4/31 (12.9%) children who had positive results, Norovirus was the most frequently identified organism, found in 3/31 (9.7%) cases. Stool cultures were obtained from 23/31 (74.1%) children; in 17/31 (54.8%), stool culture results came back negative. All 31 (100%) patients were managed conservatively with gut rest and antibiotics. The duration of gut rest varied among patients, ranging from 3 to 21 days, with a median of 9 days. A favourable outcome was observed in 25/31 (80.6%) children, whereas 6/31 (19.4%) patients (19.4%) had a poor outcome: 5/31 patients passed away, and 1 patient lost enteral autonomy and was discharged from hospital on home parenteral nutrition (HPN) (see Figure 2).

**Figure 3:**
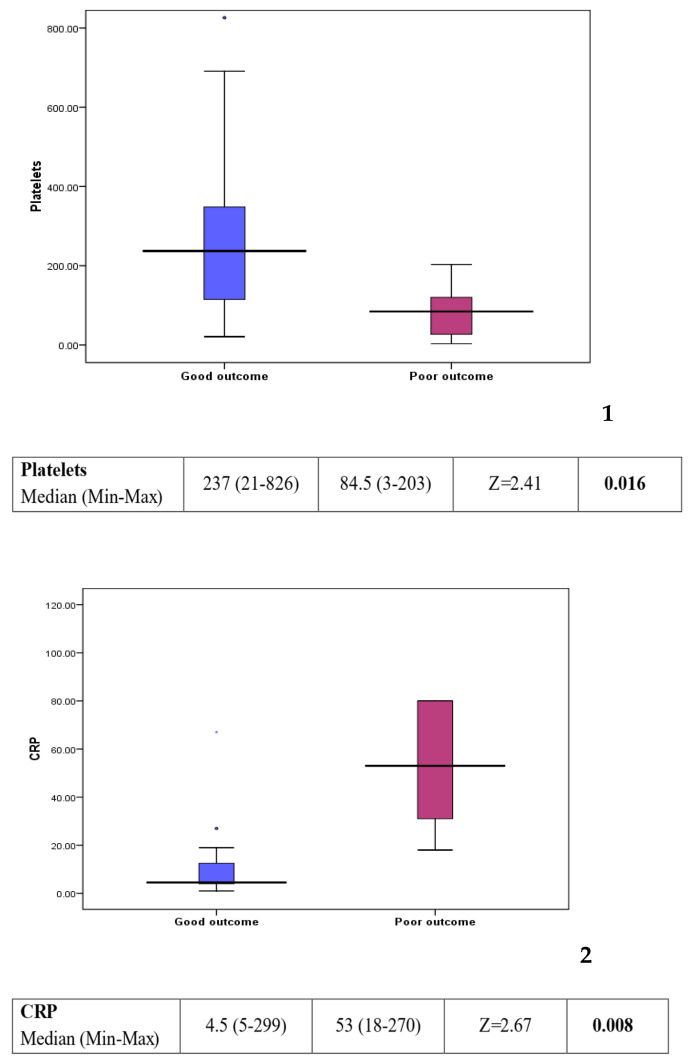

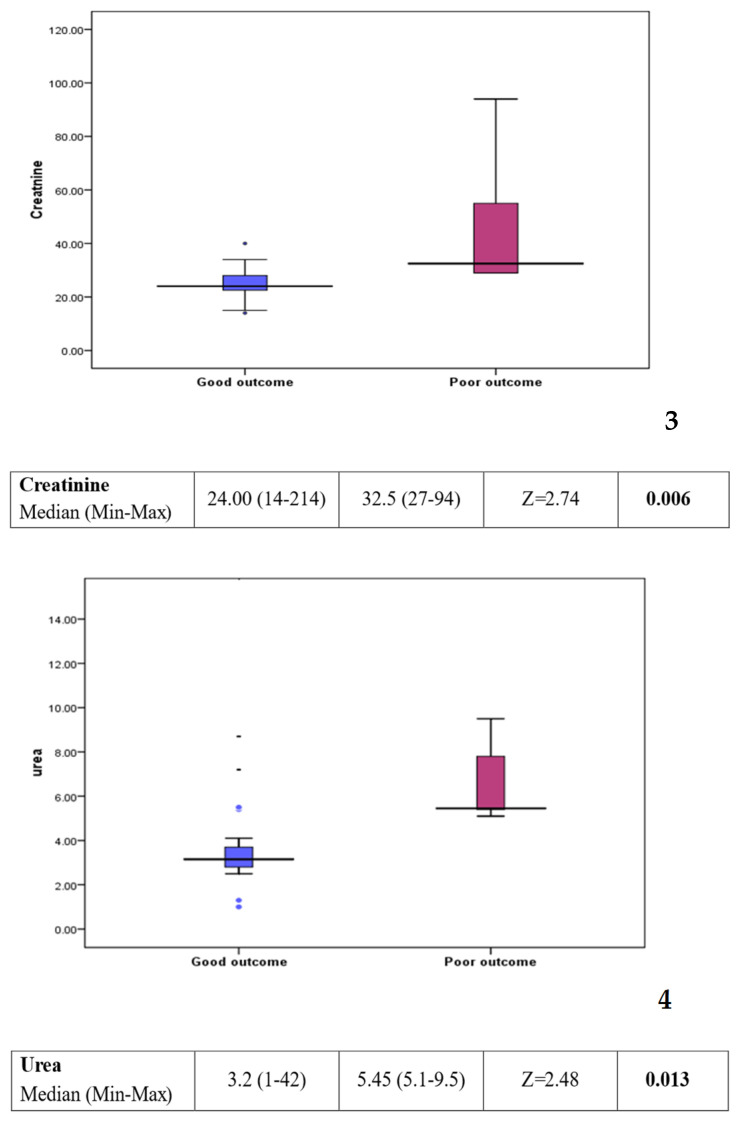
Box plots 1–4: comparison of statistically significant laboratory markers for good versus poor outcome. The subsequent box blots could be numbered individually as box plot 1: platelet could in patients with a good response compared to those with a poor outcome. Box plot 2: CRP level in patient with a good response compared to those with a poor outcome. Box plot 3: creatinine level in patients with a good response compared to those with a poor outcome Box plot 4: urea level in patient with a good response compared to those with a poor outcome.

Comparison between group 1 (good outcome) and group 2 (poor outcome) showed a statistically significant difference in some of the laboratory investigations (see Figure 3). Platelet count was found to be lower in the poor outcome group (*p* value: 0.016). On the other hand, C-reactive protein, creatinine, and urea levels were found to be higher in the poor outcome group (*p* value: 0.008, 0.006, and 0.013, respectively). Other factors, including clinical background, feeding history, and medications, showed no statistically significant difference between the two groups (see Table 2). 

## 4. Discussion

PI is a well-described radiological sign of NEC in the neonatal period, where it occurs mostly in premature infants. In children, PI is less well understood. Consensus on the clinical significance and a systematic management approach are lacking. The true incidence is unknown, as it has been an incidental finding in asymptomatic patients published in case series [6,7]. We had a similar experience, as a little over 1/3 of our children (35.5%) were found to have signs of PI on abdominal X-ray conducted for other clinical reasons. PI appears to be more common in patients with a complex medical background, reflected also in our cohort of children who developed PI whilst already admitted to hospital for inpatient therapy. Healthy children are rarely described to develop PI [8]. Clinical presentation and outcome are variable, and both asymptomatic patients and those with life-threatening symptoms have been described [8]. The majority of children in our series of 31 patients made a full recovery, but one child had prolonged enteral feed intolerance and five children died. The outcome was hence poor in one-fifth of patients and therefore relatively high. This might be explained by the complex nature and level of health needs of the patients seen in a quaternary children’s referral hospital. Predicting a poor outcome may lead to more aggressive management early after diagnosis and hence be in the patient’s interest. Furthermore, parents and carers could be more appropriately informed about what the prognosis for their child may be.

However, the underlying pathophysiology and associated risk factors for a poor outcome remain ill defined. Several theories have been proposed to explain the patho-mechanisms leading to the occurrence of air within the wall of the bowel. The bacterial theory suggests that gas-forming bacteria enter through defects in the mucosa, possibly because of underlying mesenteric ischemia [9]. Supporters of the mechanical theory argue that increased intraluminal pressure from obstruction may force gas into the bowel wall through mucosal damage. Additionally, pulmonary pathology can lead to alveolar rupture, which may result in the dissection of air along vessels and eventually reach the bowel wall [9].

Children who underwent a haematopoetic stem cell transplant, and those on immunosuppressive medication or suffering from neurodevelopmental disorders such as cerebral palsy, appear to be more prone to the development of PI, but why they have an increased risk compared to others with the same clinical background is not known.

We wanted to understand if there were defined risk factors associated with a less favourable outcome, and therefore compared patients with a good outcome to those with a poor outcome (defined as death or loss of enteral autonomy within 1 month of diagnosis of PI). There was male predominance: 67.7% of patients were boys. However, unlike the results described in a previous study [10], there was no statistical difference between male sex and good outcome or male sex and bad outcome. Gender hence appeared not to be a risk factor. The same applied for age. The median age at diagnosis of PI was 5 years (0.25–15 years). Most patients (12/31; 38.7%) were between 1 and 5 years old, but there was no association between being of a younger age and increased risk.

Unlike previous studies which focused on PI in defined groups of paediatric patients, such as post-HSCT patients, oncology patients, or intestinal failure patients [4,5,9], we included all patients who were diagnosed with PI regardless of their medical background. This allowed us to gain more insight into which children may be more prone to develop PI and what possible underlying risk factors they had. In our cohort, 16.13% of the patients had cerebral palsy, 16.13% had ALL, 12.9% had SCID, and 9.7% had received a solid organ transplant. Like previously published results, cerebral palsy and receiving immunosuppressive medication were common in children developing PI. However, whilst most of the patients had one defined medical diagnosis, some of them had more than one medical problem. There was one child with Ewing’s sarcoma on top of achondroplasia, another with HLH who developed VEOIBD, and one patient with Emmanual syndrome and Hirschsprung disease. We hence wondered if pre-existing GI pathology would make children more prone to the development of PI. Among those who had underlying GI conditions, the most common problem was gut GVHD (7 patients, 22.6%). It would hence be plausible to speculate that GVHD of the gut in a child on immunosuppression post-bone marrow or solid organ transplant may hence increase the risk to develop PI and explain why it does not occur in all children who underwent a form of transplant. However, 48.3% of our patients did not have any underlying GI conditions. GI pathology, as such, is hence not necessarily a risk factor but increases the risk of children following transplantation.

PI was an unexpected finding in more than one-third (35.5%) of our patients who had imaging for other clinical reasons whilst being asymptomatic. Compared to previous reports in the literature, where between 11 and 24.3% of asymptomatic patients were identified incidentally, our numbers were therefore higher [4,6,11,12].

The true incidence of PI amongst children with chronic health problems may hence be much greater than currently estimated. All of our patients had a long-standing medical diagnosis, and we had no comparison group of healthy peers. It is hence not possible to comment if PI occurs in healthy children.

Amongst the patients who were symptomatic at presentation, the most common symptom described was abdominal pain in just over one-fifth (22.6%) of children. This is in line with findings in previous studies, where abdominal pain was also the most common complaint in over 50% of cases, followed by abdominal distension in around 30 to 50% [4,6,7,10,13].

When comparing group 1 and group 2 in our cohort, we could not find a correlation between a presenting clinical symptom and poor outcome, including abdominal pain and/or distension. In themselves, they do hence not represent a worse prognostic factor.

The proportion of children who received enteral tube feeding in addition to, or instead of oral food intake was 58% in our cohort and therefore high. This can be explained by the complex health needs of the children studied. Three patients were already kept nil by mouth (NBM) and hence not receiving any form of oral or enteral diet at the time of PI diagnosis.

Only just over one-third (35%) of patients managed an exclusive oral diet.

We questioned if enteral tube feeding could hence be associated with a less favourable outcome and therefore be an associated risk factor. There was no statistical difference between mode of feeding and outcome between the two groups in our study. In addition, two of the three patients who were already NBM had a good outcome compared to only one child NBM with a poor outcome.

Although tube feeding was raised as a possible risk factor of PI in adult studies, we could not demonstrate the same in children [14,15].

Immunosuppressive medications, in particular steroids, have been associated with the development of PI in children [9,10,16]. It is thought that the resulting microbial dysbiosis in the gut and/or altered immune response may cause intestinal mucosal injury and, therefore, bacterial translocation and pneumatosis. On the other hand, steroid therapy was found to double the chances of PI recovery in one study [4]. In our cohort, 60% of patients with a good outcome received steroids compared to 50% with a poor outcome. The use of systemic steroids was hence not associated with a poor outcome in the majority of children.

However, when comparing patients who did well (group 1) to those with a poor outcome (group 2) worse prognosis was associated with a lower platelet count (*p* value: 0.016), raised C-reactive protein (CRP) (*p* value = 0.008), higher creatinine (*p* value = 0.006), and higher urea (*p* value = 0.013).

In contrast to the findings of Acker et al., neutropenia was not a risk factor of poor outcome in our cohort [17].

PI is often managed in a similar manner regardless of its underlying cause. Treatment typically involves a combination of strategies such as gastrointestinal rest (keeping nil by mouth), administration of antibiotics, providing hemodynamic support, and ensuring proper nutritional support for patients with parenteral nutrition [18]. All of our patients were managed in a conservative manner with gut rest and antibiotics. None of the children required a surgical intervention, even those who had a less favourable outcome. The duration of gut rest ranged from 3 to 21 days depending on the clinical condition of the child. Parenteral nutrition was used in 61.3% of the patients.

The limitations of this study are its retrospective nature, the relatively small sample size, and the complexity of the patients already receiving treatment in a highly specialised children’s hospital. Many were exposed to multiple risk factors simultaneously, including GVHD, steroid use, chemotherapy, and tube feeding. It is hence difficult to understand why some children developed PI, whilst others did not, as not one risk factor in isolation may be held responsible. However, our data confirm that PI in children appears to occur in patients with complex medical backgrounds, not in healthy children, as we had, e.g., no patients who received treatment on the orthopaedic ward for a complex fracture but were otherwise healthy children. Further studies in children presenting to a district general hospital may provide more insight, and a multicentre trial involving several hospitals would allow for more patients to be studied.

To our knowledge, this is the first study that identifies raised CRP, creatinine, and urea levels, and higher platelets as risk factors of poor outcome in paediatric patients who developed PI whilst in hospital. Potential explanations for higher inflammatory markers in these children could be worsening inflammation of the intestinal mucosa or a marker of sepsis caused by possible translocation of gut bacteria into the blood stream. Higher urea and creatinine may be due to evolving renal impairment. Regular measurement of these parameters could therefore help to alert the clinician and recognise those children who are clinically deteriorating earlier and initiate a change in management, e.g., a change in antibiotics or surgical opinion to see if a laparotomy should be considered. Families could also be more appropriately counselled about the clinical status of their child.

## 5. Conclusions

PI rarely occurs in childhood outside the neonatal period. The overall prognosis is good but associated with significant morbidity and mortality in a small number of patients. Conservative management is sufficient to achieve symptom resolution and surgical intervention was not required in our cohort. Our data suggest that lower platelet count, and higher urea, creatinine, and CRP levels are risk factors for poorer outcome, which may help guide the clinician to predict which children may not make a full recovery and therefore require long-term management, including home parenteral nutrition.

## Figures and Tables

**Table 1:** Underlying GI conditions of patients with PI.

Underlying GI Condition	The Study Group (n = 31)	
	Number	Percentage (%)
None	15	48.3
Gut graft versus host disease (GVHD)	7	22.6
Chronic constipation	2	6.5
Suspected Typhlitis	2	6.5
Ano-rectal malformation (ARM)	1	6.5
Mucositis	1	3.2
Hirschsprung disease	1	3.2
Very early onset inflammatory bowel disease (VEOIBD)	1	3.2
Colostomy	1	3.2

**Table 2:** Comparison of laboratory investigations between group 1 and group 2.

Laboratory Investigations	Favourable Outcome n = 25	Poor Outcome n = 6	*p* Value
WBCs Median (range)	5.61 (0.19–95)	5.43 (0.12–54)	0.867
Neutrophils Median (range)	2.89 (0.01–12.3)	4.03 (0.05–10.6)	0.901
Na Mean ± SD	139.61 ± 3.57	140.83 ± 2.48	0.439
K Mean ± SD	3.80 ± 0.75	4.25 ± 0.45	0.175
Ca Median (range)	2.24 (2.02–2.75)	2.43 (1.96–23)	0.083
Mg Mean ± SD	0.86 ± 0.15	0.913 ± 0.07	0.413
Lactate Median (range)	1.19 (0.67–2.6)	5.4 (5.4–5.4)	0.157
HCO_3_ Mean ± SD	26.21 ± 4.32	24.25 ± 1.34	0.563
Platelets count: Median (range)	237 (21–826)	84.5 (3–203)	0.016
C-reactive protein: Median (range)	4.5 (5–299)	53 (18–270)	0.016
Serum Creatinine: Median (range)	24.00 (14–214)	32.5 (27–94)	0.006
Serum Urea: Median (range)	3.2 (1–42)	5.45 (5.1–9.5)	0.013

## Data Availability

The original contributions presented in this study are included in the article. Further inquiries can be directed to the corresponding author.
